# Audit-as-code: a policy-as-code framework for continuous AI assurance

**DOI:** 10.3389/frai.2026.1759211

**Published:** 2026-02-26

**Authors:** Aoun E. Muhammad, Kin-Choong Yow, Shrooq Alsenan

**Affiliations:** 1 Faculty of Engineering and Applied Science, University of Regina, Regina, SK, Canada; 2 Department of Information Systems, College of Computer and Information Sciences, Princess Nourah Bint Abdurahman University, Riyadh, Saudi Arabia

**Keywords:** AI assurance, CI/CD, compliance, explainability, governance, policy-as-code, reproducibility, traceability

## Abstract

**Introduction:**

Existing AI assurance and governance frameworks rely heavily on documented written policies and manual reviews of the implementation. The primary challenge is not the length of these documents, but to operationalize the gap from transforming qualitative requirements into verifiable controls. This approach makes ensuring continuous compliance through the development life cycle hard to enforce, scale, and reproduce.

**Methods:**

This study presents a continuous assurance framework called Audit-as-Code that maps governance requirements to technically-auditable rules, that can be a combination of versioned policy specification and executable checks for evidence artifacts, linked to structured evidence regarding data, models, provenance, performance, decisions, and explanations regarding the decisions being made. While the framework addresses the governance and regulatory mapping requirements, the primary focus of this study is MLOps/CI-CD (continuous integration/continuous delivery) operationalization, and turning these requirements into deterministic checks and gate decisions integrated in operational workflows. In this study, we introduce an assured readiness score that integrates the governance risk with other key responsible AI principles, such as traceability and explainability. This approach helps in aligning deployment decisions with predefined risk tiers, and the framework automates decisions on whether a system can proceed, requires remediation and fixes, or should be blocked. It also provides targeted suggestions for improvement and compliance for the lags identified.

**Results:**

We evaluated this framework on representative AI systems and demonstrated how a single evidence bundle can be used to support assessment across different governance regulations.

**Discussion:**

In doing so, Audit-as-Code ensures AI assurance transforms from a documentation-driven policy module to a quantitative, auditable, reproducible, and operationally practical module to ensure compliance.

## 1 Introduction

As artificial intelligence (AI) systems are built and deployed, the creation and use of AI systems will need to conform to laws and regulations, providing the expectation for transparency, traceability, and human supervision. For example, given a deployed prediction, the notion of traceability means there is an ability to recover the exact model hash and training run, along with the dataset split and preprocessing lineage. And the associated audit trail is established that links the prediction to its explanation. Currently, the laws and regulations establish governance by outlining and providing principles and rules to serve as a guide and limitations on how AI systems can be developed and used. The governance guidelines provided by governing bodies are critical in the governance of AI systems. However, they inherently have a structural misalignment with the software engineering process and workflow, which require particular criteria such as executable criteria, reproducible evidence, and deterministic acceptance gates. As a result, this leads to compliance outcomes that are fragile, audit tracks that are inconsistent, and regression detection will occur with delays, long after the actual version change has taken place within an AI model, data, and/or configuration. For example, many governance obligations can be satisfied through periodic, point-in-time documentation reviews that include conducting risk and impact assessments, and transparency reports. Whereas modern AI development is iterative and change-driven, where models, datasets, prompts, configurations, and dependencies can be changed, updated, and/or modified across frequent pull requests and releases. This dynamic and iterative change environment creates gaps like (i) reviews becoming stale relative to the deployed artifacts, (ii) manual collection of evidence compared to automated integration as part of the build pipeline, and (iii) post-deployment monitoring and change-control requirements being treated as operational “afterthoughts” rather than enforced release criteria. Our proposed framework addresses these gaps by encoding such obligations as versioned and verifiable gate criteria, and re-evaluated under change control.

In this study, we propose Audit-as-Code, a continuous assurance approach that maps governance requirements as quantifiable executable artifacts in the secure software development lifecycle. At its most basic level, Audit-as-Code maps the governing policies and operationalizes them into small and versioned specifications. It binds the defined and approved policies to a machine-checkable evidence bundle output produced by lightweight collectors. And evaluates the policy and evidence bundle through a non-interactive gate in continuous integration/continuous delivery (CI/CD). This gate produces deterministic, quantifiable *PASS/WARN/BLOCK* decision-making output, along with minimal human-readable Fix-It guidance. The Fix-It guidance specifies the fixes and remediation for the causes of Warn/Block decisions, and provides reasoning for the Warn/Block decision. This framework tries to ensure governance regulations are mapped from theoretical/in-principle guidelines to ad-hoc quantifiable interpretations that are explainable, accountable, traceable, and reproducible.

Audit-as-Code calculates assurance of a developed model along two distinct quantifiable dimensions, namely: traceability index (TI), and explainability index (XI). Traceability measures whether a developed AI model has inputs, code, models, and decisions that are traceable, verifiable, and reliably reproducible through provenance (e.g., dataset versioning, model versioning, pipeline log traceability, decision audit trails, and empirical replication). On the other hand, explainability evaluates whether the developed AI model is capable of providing explanations that are accurate, stable, reproducible, and reasonable, as well as usable by their intended end users. Both axes are normalized to [0, 1] using observable maturity bands (Minimal → Critical) with adjustable component weights for domain specificity. To summarize operational readiness, while retaining the weakest pillar, we calculate an Assured Readiness Score (ARS) as follows:


$$\begin{array}{l} \text{ARS}=\sqrt{\text{Risk}\cdot \min\left(\text{TI},\text{XI}\right)}, \end{array}$$ (1)

where *Risk* is derived from applicable Governance frameworks with optional jurisdictional overlays. The deployability does not depend solely on the ARS score. Under Audit-as-code, a developed AI model is eligible for production-level deployment only if both TI and XI meet *tier-specific thresholds*. We assume Risk ∈ [0, 1] as a normalized composite governance risk view used for tiering. The square-root is a monotone concave transform that preserves ordering while reducing dominance of extreme risk values in the scalar summary, keeping ARS ∈ [0, 1] and improving interpretability for triage and comparison.

Audit-as-Code differentiates between the quantification of risk scoring from the eligibility of the model to be deployed. The ARS indicates where a model lies on an operational risk spectrum (sandbox → limited use → pilot → deploy with controls → deploy with audits). Here, operational risk spectrum denotes a set of policy-defined *deployment modes* associated with ARS bands (Table 3) rather than a qualitative description of risk. Lower bands restrict release to non-production contexts like for sandbox or limited use). The Intermediate bands permit only controlled pilot rollouts with mandatory reviews and remediations. And the higher bands allow production deployment with additional safeguards, such as deploy-with-controls or continuous auditability requirements, such as deploy-with-audits. These modes apply only after eligibility checks like tier minima and safety blockers are satisfied. While an easy-to-comprehend, transparent tier table enforces non-negotiable minima for TI and XI indices per risk band (minimal, low, moderate, high, critical). In addition to the criticality thresholds, the policies can declare blockers and framework-agnostic safety requirements with clear operational impacts, e.g., maximum or minimum fairness threshold, no personally identifiable information (PII) leakage into model inputs/outputs (I/O), and/or bounds on residual risk under red-team (adversarial) probes. These policies can automatically yield BLOCK regardless of averages or risk-quantified scores. This dual-check hierarchy prevents strong performance on one pillar from weaknesses-obfuscation on the other, and makes it virtually impossible to average out safety violations.

The proposed framework consists of four operational components:

1. A policy file which includes and encodes tier thresholds, ARS bands, and critical safety-blockers;
2. A bounded evidence schema, which produces versioned JSON artifacts for lineage, replication;
3. A gate engine that checks inputs against the boundaries and ranges defined in schema executes validation checks [e.g., CI (Continous Integration) scripts such as Python/SQL checks], calculates TI, XI, and ARS, applies thresholds and critical safety-blockers, and
4. A reviewer dashboards that visualize readiness metrics [risk vs. min(TI, XI)], tier classification, component breakdowns, and critical safety-blockers. The same deployment gate is used in continuous integration/continuous delivery (CI/CD) as a non-interactive task.

The reproducibility is ensured by recording the seeds in use, referencing the version of the container and Python being used, and the specific version of the policy being enforced. Hashing (for computational components) and attestations of input artifacts assist in adding an extra layer of assurance to the reproducibility. Because the policy is versioned, any changes to thresholds, bands, or blockers will deterministically impact outcomes, and the changes can be reviewed as a code difference. In the UI, we display jurisdictional overlays, e.g., EU-AI Act (European Parliament and Council, 2024), NIST RMF (National Institute of Standards and Technology, 2023), ISO27001/ISO42001, that have an impact on the thresholds definition and appetite of the risk matrix of the model.

We evaluated Audit-as-Code over synthetic and real-time scenarios, and developed a synthetic corpus that spans multiple modalities like Large Language Models (LLM), CV, tabular, and time series. And corresponding risk tiers to demonstrate how assured readiness is deployed via a strict policy and how fallback ablation enhances the *PASS* outcomes for near-miss cases without altering the ARS band definitions.

**Contributions**.

The key contributions of our study are as follows:

1. A policy-as-code framework with risk tiers and implementing safety blockers, a tier-mapped evidence schema. And a deterministic gate that runs both in continuous integration/continuous delivery (CI/CD) and during audits, and provides *PASS/WARN/BLOCK* decision with Fix-It remediation guidelines.
2. An assured readiness score calculated via $\text{ARS}=\sqrt{\text{Risk}\cdot \min\left(\text{TI},\text{XI}\right)}$ for operational mapping of risk, traceability, and explainability to the compliance requirements enforced by tier specific minima to ensure averaging effect do not obfuscate the weak axes.
3. Tunable jurisdictional overlays for risk and ARS scoring while keeping thresholds invariant, and enabling cross-framework comparisons with the same batch of evidence.
4. Seeds, policy, code versioning, and optional artefact hashing and attestation ensure that outcomes generated are auditably reproducible across runs, re-runs, and environments.
5. Corpus-scale evaluation to see the effectiveness and relevance of assured readiness score (ARS). And penalizing systems for missing evidence, blockers, and threshold violations. Also, conducted audits on real-world models while demonstrating minimal, targeted remediation that helps in evaluating and fixing the lags in compliance issues across frameworks without compromising threshold requirements.

**Figure 1:**
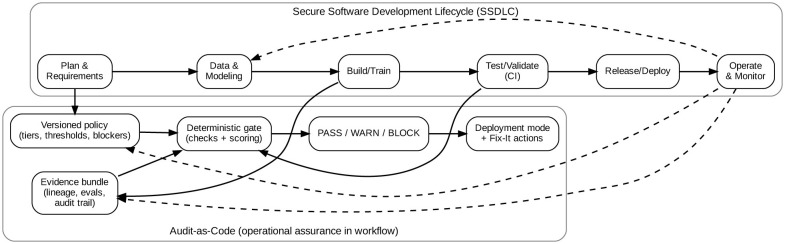
Audit-as-code positioned within a typical secure software development lifecycle (SSDLC).

Contrary to approaches like Model Cards and FactSheets that emphasize documentation, our contribution is a quantifiable, enforceable mechanism with a bounded, schema-checked evidence bundle, a deterministic Gate that caters to traceability and explainability to conform to tiered, policy-aligned decisions with *PASS/WARN/BLOCK*. Figure 1 situates Audit-as-Code within standard secure software development lifecycle (SSDLC)/CI-CD workflow for operational mapping. Audit-as-Code presented in this study is implemented as a reference pipeline for the reported experiments and evaluation, not as an out-of-the-box production-ready utility. Practical deployment may require configuration and integration adjustments to fit the target organization's technology stack and production environment. This study provides a proof-of-concept showing where and how Audit-as-Code can be embedded in SSDLC/CI-CD workflows to support responsible AI release decisions.

## 2 Background and related work

Most of the AI governance frameworks focus on risk management, transparency, traceability, robustness, accountability, and human auditing. And they purposefully provide guidance that is technology-agnostic on how those objectives can be accomplished. This leaves the implementation of the guidelines up to practitioners, engineers, and auditors on how to specify the controls that will be enforced, the types of evidence needed for evaluations and making final determinations, the evidence verification, and non-conformances/violations that will result in deployment block actions. The EU AI Act (European Parliament and Council, 2024) provides detailed guidance and principle obligations as per the use of AI in specific domains, but it still allows discretion in how to enforce the guidelines technically. NIST's AI RMF (National Institute of Standards and Technology, 2023) classifies the activities in four stages, i.e., MAP, MEASURE, MANAGE, and GOVERN, but it does not specify any stringent thresholds. ISO/IEC 42001 (International Organization for Standardization and International Electrotechnical Commission, 2023b) and 23894 (International Organization for Standardization and International Electrotechnical Commission, 2023a) describe the requirements for managing risks related to AI development, systems, and how the risk should be treated, but do not explicitly specify how these requirements should be implemented. OECD/UNESCO (Organisation for Economic Co-operation and Development, OECD; United Nations Educational, Scientific and Cultural Organization, UNESCO) focuses on human-centric decisions and accountability of AI models, and ethical principles to observe while developing AI systems. ICO (Information Commissioner's Office and The Alan Turing Institute, 2020) and FDA/IMDRF (U.S. Food and Drug Administration, FDA) refer to the mandated documentation and requirements for safe development and deployment of AI systems. This research focuses on the pipeline-driven “how” specifications by binding these governance guidelines to a quantifiable and verifiably-bounded, machine-readable evidence schema and executing deterministic checks for tiers, thresholds, and blockers in continuous integration/continuous delivery (CI/CD).

### 2.1 Policy-as-code and domain specific practices

Policy-as-Code (PaC) deals with conformance through declared rules enforced inherently via gatekeepers. The PaC plays a sort of similar role as in other as-a-code frameworks in cloud/supply-chain contexts—including Open Policy Agent (Open Policy Agent Project, 2021) and Regulation-based policy as code, provenance, and ensure integrity. This demonstrates the value of combined code-defined rules and their associated attestations for evidence-based reproducibility and traceability (OpenSSF, 2023). On the other hand, documentation-dominated regulations are the default means of ensuring compliance with requirements in AI programs (Information Commissioner's Office and The Alan Turing Institute, 2020; National Institute of Standards and Technology, 2023; International Organization for Standardization and International Electrotechnical Commission, 2023b), which creates a significant gap between declared policy requirements and controls that are implemented in a deterministic way through continuous integration/continuous delivery (CI/CD) pipelines.

The concept of algorithmic auditing refers to ensure fairness, safety, documentation sufficiency, identifying and treating operational risk management across the full development life cycle. However, anomalies identified during real-time scans and implementation of industry-level good practices show the importance and effectiveness of clearer role definitions, segregation of duties, rigor in methodology, and independent audit claims (Costanza-Chock et al., 2022; Slattery et al., 2024; Koshiyama et al., 2024).

The gap research conducted in the financial sector provides an example of how projects with a nation-wide scope are continuously at risk and have a continuous need for monitoring, oversight, and risk controls (Bracke et al., 2019; Tallberg et al., 2023).

The recent research studies continue to present findings such as biases in the data/process, insufficient documentation, and auditor variability (Bandy, 2021; Eitel-Porter, 2021; Shen et al., 2021), stressing the work we must do to operationalize principles to convert them into executable criteria with determinative gates.

### 2.2 Technical assurance axes: traceability and explainability

the relevant research regarding the two main components of this research (Traceability and Explainability) is as follows:

- **Traceability (TI)** is the degree to which an AI system's outputs and decisions can be reconstructed and verified end-to-end across the development lifecycle. This comprises tracing from the producing input context, the exact data, code, configuration, and model version used, to the resulting predictions or decisions made, and the logged outcomes. This can be accomplished using provenance techniques, integrity-protected artifacts like lineage metadata, audit trails, and reproducibility records. A part of earlier literature simplifies the concept of traceability to what “good” looks like across artifacts (dataset/model versions, logs/audit trails, replication) and identifies common maturity gaps (Mora-Cantallops et al., 2021). Operational tools like MLflow and Data Version Control (DVC) are used to capture lineage and executable runs, for experiment and artefact versioning and reproducibility of pipelines (Zaharia et al., 2018; Iterative.ai, 2018). Observational Medical Outcomes Partnership (OMOP) driven imaging pipelines are used to achieve alignment of clinical metadata with model outputs (Park et al., 2024). (Namlı et al. 2024) illustrates transparent, replayable processing in end-to-end health data solutions. Some researchers stress the need for an immutable evidence/decision trail, and machine-readable identifiers for evidence gathering and analysis (Fraga et al., 2024; Gobin et al., 2025). Finally, studies related to practically mapping traceability to reliability under real-time scenarios with operational constraints such as health and safety contexts where auditability and replication materially impact risk are discussed in (Taimoor and Rehman 2022) and (Chaminade et al. 2012).
- **Explainability (XI)**. Explainability is the degree to which an AI system provides decision explanations that are *faithful* to the model's actual reasoning signals rather than post-hoc narratives. These explanations are expected to be *stable* and *robust* under small input perturbations, and *usable* for the intended users and decision context. Experimental methodologies provide different lenses, such as local surrogates (LIME) and additive feature correlations (SHAP), which are used for tabular/text (Ribeiro et al., 2016; Lundberg and Lee, 2017) explanations. The gradient-based visual localization for deep vision models is discussed in (Selvaraju et al. 2017). In Doshi-Velez and Kim (2017) and (Zhang et al. 2022), it is demonstrated how measurable standards like fidelity, faithfulness, stability, and robustness are used to add to explainability and comprehensibility to the intended user. In (Holzinger et al. 2019) and (Buçinca et al. 2020), authors warn against overly relying on explanations that are post-hoc and not quantifiable. Human comprehensibility, on the other hand, discusses causability and task-specific explanations that should assist operators, engineers, auditors, and upper management in making safer and relatively correct decisions, and not simply explanations that seem plausible. In (Okada et al. 2023) and (Minh et al. 2021), authors discuss how domain surveys/reviews attempt to translate explainability into applicable settings. They also emphasized how stability and usability requirements are important beyond just visual saliency. In (Maloca et al. 2024) and (Wachter et al. 2017), authors discuss how empirical artifacts are better suited for explanations compared to the explanation pipelines that can be misleading, such as selection bias or better-suited ground-truth effects. They suggested robustness and coverage checks in CI rather than a hastily constructed dashboard.

Core XAI methodologies, including LIME (Ribeiro et al., 2016), SHAP (Lundberg and Lee, 2017), and Grad-CAM (Selvaraju et al., 2017), provide both local and/or global explanations across various modalities. A durable conclusion intrinsic to the literature is to assess explanation quality means at minimum some kind of quantitative assessment—parameters like fidelity to model behavior, stability across the model and causal faithfulness, robustness to perturbation, coverage/logging—and assessment of human comprehensibility for decision making, especially in high-risk, high-stakes scenarios (Doshi-Velez and Kim, 2017; Holzinger et al., 2019; Zhang et al., 2022; Buçinca et al., 2020; Okada et al., 2023; Minh et al., 2021; Maloca et al., 2024; Wachter et al., 2017).

To ensure development and deployment of a robust AI, traceability and replicability are very critical parameters to foster trust and confidence in the trustworthiness of decisions and explanations provided. The governance regulations and industry guidelines emphasize on the importance of dataset/model versioning, logging of the pipeline, continuous monitoring, audit trails, and reproducible protocols (Mora-Cantallops et al., 2021; Park et al., 2024; Namlı et al., 2024; Fraga et al., 2024; Gobin et al., 2025; Taimoor and Rehman, 2022). Tools (MLflow, DVC) can support the operational versioning and documentation requirements, but as the surveys indicate, this approach is still qualitative rather than being a quantifiable approach, and does not provide any comparable maturity tiers and indexes (Zaharia et al., 2018; Iterative.ai, 2018; Mora-Cantallops et al., 2021).

Furthermore, relevant governance regulations also discuss the importance of traceability and how it can support verifiability and accountability for developed models (Wachter et al., 2017; Papadimitriou, 2023; Information Commissioner's Office and The Alan Turing Institute, 2020).

### 2.3 Documentation-centric governance and practical auditing

Model Cards (Mitchell et al., 2019) and AI FactSheets (Arnold et al., 2019; Gebru et al., 2021) present needs and ways to accomplish transparency by normalizing information about model configurations, data specifications, and configurations, and post-hoc evaluations. Although they advocate for consistent reporting, these tools do not provide quantitative metrics of robustness, faithfulness, and human comprehensibility, nor do they enforce automated compliance or sufficiency checks. The documentation-only approaches leave a considerable gap in the implementation of the assurance matrix for untested scenarios. Documentation may generate visibility, but insufficient implementation for definite remediation decisions (Mitchell et al., 2019; Arnold et al., 2019; Koshiyama et al., 2024; Slattery et al., 2024).

In (Langer et al. 2021) and (Speith 2022), authors proposed an interdisciplinary approach for explainability auditing that emphasizes developing a standardized criterion for formats for evidence, and segregation of roles. Studies in the health and finance sectors also propose causability, end-user comprehensibility, and transparency for AI systems (Holzinger et al., 2019; Okada et al., 2023; Zhang et al., 2022; Bracke et al., 2019).

Also, researcher have emphasized on the pragmatic realities of a trustworthy AI, observations regarding scalable and quantifiable governance (or lack thereof) for small entities, and whether organizations developing AIs and potentially integrated data ecosystems, will need to develop internal audit functions (Sanderson et al., 2021; Crockett et al., 2023; Schuett, 2024). Environmental, Social, and Governance (ESG) and sector-specific studies cater for stakeholders' confidence, expectations and professional codes of ethics needed for audit mechanisms and continuous monitoring (Minkkinen et al., 2022; Hasan et al., 2024). Finally, in (Li et al. 2023), authors emphasized the principles and practices of collecting evidence that is needed for the implementation of trustworthy AI.

Recent policy and governance initiatives have emphasized the operational and lifecycle assurance for AI systems. This includes continuous oversight, reporting, and post-deployment monitoring of the AI systems. In the United States, OMB Memorandum M-24-10 (Office of Management and Budget, 2024) directs federal agencies to strengthen AI governance and risk management practices reflecting the focus on institutionalized and continuous oversight. The 2024 Seoul AI Summit (Ministry of Foreign Affairs of Japan, 2024) and accompanying Frontier AI Safety Commitments (UK Government, 2024) have elevated expectations for AI safety, coordination, and highlighted practices like red-teaming, public reporting of capabilities and limitations, and safeguards prior to release. Similarly, the G7 Hiroshima AI Process (OECD, 2024) proposed a Code-of-Conduct reporting framework and standardized disclosures to ensure organizational accountability. Australia's Voluntary AI Safety Standard (Department of Industry, Science and Resources (Australia), 2024) provides actionable guardrails and implementation guidance for organizations.

## 3 Methodology

AI Audit-as-code can be defined as a set of actionable policies in the form of machine-checkable evidence. These policies are enforced via a deterministic gate that runs either locally or in continuous integration/continuous delivery (CI/CD). The traceability (TI) and explainability (XI) indices are calculated by scoring verifiable artifacts and comparing them against tier thresholds defined by the governance Risk view. The Assured Readiness Score (ARS) summarizes the overall posture of risks involved, but it does not override or compromise the tier thresholds and safety blockers. All inputs, thresholds, and outcomes are stored as versioned artifacts to ensure reproducibility. In Audit-as-Code, “technically-auditable rules” are implemented as (i) a versioned policy specification [e.g., **Y**AML **A**'int **M**arkup **L**anguage (YAML), a human-readable configuration format, or JSON] encoding tier thresholds, weights, and non-overridable blockers; (ii) evidence schema constraints defining the required structure of the evidence bundle artifacts; and (iii) executable validation checks (e.g., continuous integration/continuous delivery (CI/CD) jobs implemented as Python validation scripts and/or SQL checks over logged evidence tables) that deterministically evaluate the evidence against the policy. These checks produce explicit PASS/WARN/BLOCK outcomes with traceable justifications and remediation deltas.

### 3.1 Risk scoring and aggregation

We calculate the risk scoring criteria used to derive both audit decisions and the Assured Readiness Score (ARS) following a utility-transformed severity core with contextual overlays (Muhammad et al., 2025). It is defined as normalized likelihood *L* ∈ [0, 1] and impact *I* ∈ [0, 1]. Here, *L* and *I* denote the normalized likelihood and impact of governance-relevant harm/failure scenarios for the system in its intended operational context (e.g., safety, rights, privacy, or material decision errors), as represented by the selected risk profile and optional jurisdictional overlays. The baseline severity is calculated as:


$$\begin{array}{l} U\left(L,I\right)=1-e^{-k\left(L\cdot I\right)}, \end{array}$$ (2)

where the curvature parameter *k*>0 controls risk aversion (we use *k* = 3 by default).

Following this, five modifiers namely Context (*C*), Governance tier (*G*), Technical surface (*T*), Environmental exposure (*E*), and Residual risk (*R*) are applied to get a final bounded composite score:


$$\begin{array}{r} S=\alpha U\left(L,I\right)+\gamma C+\delta G+\theta T+\text{λ}E+\rho R,\\ \alpha +\gamma +\delta +\theta +\text{λ}+\rho =1. \end{array}$$ (3)

Within the deployed Gates, *blockers* are enforced on selected dimensions (e.g., fairness gap and PII) as well as on residual risk. For example, at the High tier we require the residual risk to be lower than the risk appetite defined by the governance steering committee *R* ≤ 0.25. thresholds on traceability (TI) and explainability (XI) are applied independently of *S*. The readiness score (ARS) is reported alongside *S* , integrated and classified into bands using fixed thresholds, as described in the study.

**Figure 2:**
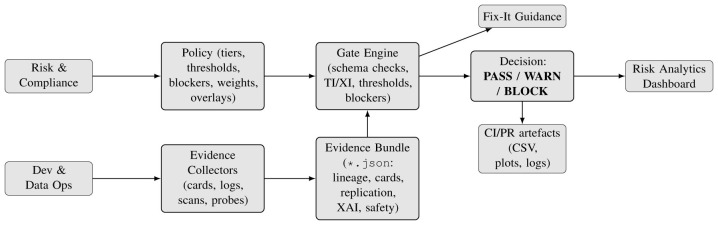
Architecture: policy and a schema-checked evidence bundle are evaluated by a deterministic gate to produce *PASS/WARN/BLOCK* outcomes, Fix-It guidance, and reviewer dashboards.

Figure 2 summarizes the Audit-as-Code architecture, showing how a versioned policy and schemachecked evidence bundle are evaluated by a deterministic gate to produce PASS/WARN/BLOCK outcomes and remediation guidance.

### 3.2 Notation and normalization

In our proposed framework, Risk is a composite governance-level view used to determine the applicable tier, and baseline deployment posture. Additionally, technical assurance is calculated via evidence components. We quantify traceability and explainability by aggregating component-level evidence signals into *TI* and *XI* on [0, 1]. The deployment gate then enforces tier-specific minima on (*TI, XI*) and any safety blockers, while ARS provides a compact summary by combining the tiered risk view with the limiting assurance dimension min(*TI, XI*).

Both the indices cater for banded components with weights that sum up to one. With component scores *s*_*c*_ ∈ [0, 1] and weights *w*_*c*_≥0 ($\underset{c}{\sum }w_{c}=1$):


$$\text{TI}=\underset{c\in \left\{\text{DV},\text{MV},\text{PL},\text{AT},\text{RR}\right\}}{\sum }w_{c}s_{c},\text{ XI}=\underset{m\in \left\{\text{LF},\text{GF},\text{FA},\text{RS},\text{CL},\text{HC}\right\}}{\sum }w_{m}s_{m}.$$

Band scores are calculated by evaluating the evidence mapped to maturity bands in the following categories: Minimal, Low, Moderate, High, Critical. We operationalize traceability index (TI) and explainability index (XI) as evidence-driven components that are quantifiably scored on the scale of [0, 1] and checked within the continuous integration/continuous delivery (CI/CD) pipeline. The TI index caters to the traceable, reproducible, and verifiable end-to-end pathway of evidence of data provenance, code versioning, and decision-making criteria. XI, on the other hand, adds value to the trustworthiness and responsibility in the deployed model by adding rationale explanations that are accurate, stable, and comprehensible for the intended users. These two indices map directly to governance expectations around transparency, documentation availability, and auditability.

In this study, we use traceability to ensure *reconstructable decision lineage* for any produced output or a decision. An auditor should be able to answer “what produced this outcome and can we reproduce or verify it under similar conditions?” by following an integrity-protected chain from the input context, to the exact data, code, configuration, and model version used, to the explanation. The following five components operationalize this notion by covering (i) data provenance and linkage (DV), (ii) model provenance (MV), (iii) run/pipeline provenance (PL), (iv) integrity-protected end-to-end audit trail (AT), and (v) reproducibility/replication checks (RR).

To evaluate the TI aspect of a model properly, explicit availability of artifacts and schema checks are needed, such as:

1. DV–data provenance and linkage refers to immutable dataset identifiers/versions (e.g., hashes) plus the metadata needed to trace an output/decision to the relevant data split/slice and preprocessing lineage; dataset versioning alone is treated as necessary but not sufficient;
2. MV–model versioning denotes each trained model with a unique hash, seeds, environment lockfiles, and a backward-compatible link to the corresponding data set IDs;
3. PL–pipeline logging refers to retaining configurations, metrics, and artefact URIs, and runtime metrics such as container image, package locks in a way that a specific run is reproducible when provided these exact details;
4. AT–audit trail refers to retaining a tamper-proof chain from input → model/version → explanation → decision → outcome; and
5. RR–reproducibility and replication refers to creating a seeded re-run with tolerances and reports on *PASS* or *FAIL* rates and variances included.

The artifacts and evidence needed to quantify XI can be divided into:

1. LF–local fidelity refers to the measurement of surrogate fidelity or attribution reconstruction error on held-out samples.
2. GF–global stability refers to quantifying the correlation/overlap of attributes across retrains.
3. FA–faithfulness refers to evaluating that highlighted features causally affect predictions via deletion/insertion curves.
4. RS–robustness refers to checking agreement of explanation/prediction under small input perturbation.
5. CL–coverage and logging refers to catering fraction of decisions with stored explanations and linked context; and
6. HC–human comprehensibility refers to a lightweight grounded rubric to evaluate time-to-insight, calibration, clarity, utility, and decision quality for intended users.

### 3.3 Maturity bands and evidence anchors (TI and XI)

The assurance readiness consists of two composite indices, namely: the Traceability Index (TI) and the Explainability Index (XI). These indices are operationalized from principle to practice by taking the form of evidence-driven weighted averages of the individual components normalized on the scale of [0, 1] interval. For TI in Table 1 and XI in Table 2, the components and thresholds of the bands are defined to be used for the audit purposes of the artifacts and evidence without subjective reinterpretation.

**Table 1:** Scoring bands for Traceability Index (TI) components.

**Component**	**Score band**	**Tier**	**Condition/example**
Dataset versioning (DV)	0.0–0.2	Minimal	No dataset versioning; ad hoc raw files.
	0.3–0.4	Low	Partial tracking; manual naming.
	0.5–0.6	Moderate	Git/DVC hashes but inconsistent.
	0.7–0.8	High	Persistent IDs; consistent hashing.
	0.9–1.0	Critical	DOI-registered datasets with full provenance.
Model versioning (MV)	0.0–0.2	Minimal	No checkpoints/versioning.
	0.3–0.4	Low	Occasional checkpoints.
	0.5–0.6	Moderate	Version tags; limited metadata.
	0.7–0.8	High	Documented releases; archived checkpoints.
	0.9–1.0	Critical	Model cards plus full archival compliance.
Pipeline logging (PL)	0.0–0.2	Minimal	No logs recorded.
	0.3–0.4	Low	Partial logs; missing seeds/configs.
	0.5–0.6	Moderate	Logs exist but incomplete.
	0.7–0.8	High	Comprehensive logging (MLflow/W&B).
	0.9–1.0	Critical	Automated, tamper-resistant pipeline logs.
Audit trail (AT)	0.0–0.2	Minimal	No decision/event logs.
	0.3–0.4	Low	Basic logs (timestamps only).
	0.5–0.6	Moderate	Logs exist but not integrity-protected.
	0.7–0.8	High	Secure logging; full trace metadata.
	0.9–1.0	Critical	Immutable, tamper-proof audit chain.
Reproducibility replication (RR)	0.0–0.2	Minimal	Replication not attempted or always fails.
	0.3–0.4	Low	≤ 30% success rate.
	0.5–0.6	Moderate	50%–70% replication success.
	0.7–0.8	High	≥80% replication success (internal/external).
	0.9–1.0	Critical	100% reproducibility under replication protocol.

Each component is normalized to [0, 1]. Values may be adjusted for sectoral or regulatory contexts.

**Table 2:** Scoring bands for Explainability Index (XI) components.

**Component**	**Score band**	**Tier**	**Condition/example**
Local fidelity (LF)	0.0–0.2	Minimal	Explanations diverge completely (*R*^2^ < 0.2).
	0.3–0.4	Low	Weak approximation (*R*^2^≈0.3).
	0.5–0.6	Moderate	Moderate fidelity; partial misalignments.
	0.7–0.8	High	High fidelity (*R*^2^≥0.7).
	0.9–1.0	Critical	Near-perfect fidelity across instances.
Global stability (GF)	0.0–0.2	Minimal	Rankings unstable (ρ < 0.2).
	0.3–0.4	Low	Weak stability (ρ≈0.3).
	0.5–0.6	Moderate	Moderate consistency across runs.
	0.7–0.8	High	Stable (ρ≥0.7) across retrainings.
	0.9–1.0	Critical	Fully stable across environments.
Faithfulness (FA)	0.0–0.2	Minimal	Importance not predictive of output.
	0.3–0.4	Low	Weak correlation.
	0.5–0.6	Moderate	Deletion AUC ≈0.5–0.6.
	0.7–0.8	High	Deletion AUC ≈0.7–0.8.
	0.9–1.0	Critical	Perfect causal alignment.
Robustness (RS)	0.0–0.2	Minimal	Explanations flip with perturbations.
	0.3–0.4	Low	Sensitive to noise.
	0.5–0.6	Moderate	Mostly stable.
	0.7–0.8	High	Robust to benign noise.
	0.9–1.0	Critical	Adversarially stress-tested; stable.
Coverage and logging (CL)	0.0–0.2	Minimal	Explanations rarely generated.
	0.3–0.4	Low	Partial coverage (< 50%).
	0.5–0.6	Moderate	Coverage ≈50%.
	0.7–0.8	High	≈80% coverage; logged.
	0.9–1.0	Critical	100% coverage; secure logging.
Human comprehensibility (HC)	0.0–0.2	Minimal	Unusable to target users.
	0.3–0.4	Low	Analysts require high effort.
	0.5–0.6	Moderate	Usable, but slow/error-prone.
	0.7–0.8	High	Improves user task accuracy/speed.
	0.9–1.0	Critical	Validated in usability studies.

Each component is normalized to [0, 1]. Values may be adapted for critical domains where fidelity and comprehensibility must be stricter.

**Table 3:** Risk tiers, minimum thresholds, and nominal decisions.

**Risk tier (by score)**	***TI*_min_**	***XI*_min_**	**Nominal decision**
Critical (≥0.85)	≥0.80	≥0.75	Deploy with audits
High (0.70–0.84)	≥0.70	≥0.70	Deploy with controls
Moderate (0.50–0.69)	≥0.60	≥0.60	Pilot/controlled rollout
Low (0.30–0.49)	≥0.50	≥0.50	Limited/internal use
Minimal (< 0.30)	–	–	Research sandbox

Unless a sectoral context is applied, TI and XI are computed with equal weights where *w*_DV_ = *w*_MV_ = *w*_PL_ = *w*_AT_ = *w*_RR_ = 0.20, and *w*_LF_ = 0.20, *w*_FA_ = 0.20, *w*_GF_ = *w*_RS_ = *w*_CL_ = *w*_HC_ = 0.15. Let *c*_*k*_ ∈ [0, 1] denote the score of component *k* as supported by its companion JSON artefact; the indices are $\text{TI}=\underset{k}{\sum }w_{k}c_{k}$ and $\text{XI}=\underset{k}{\sum }w_{k}c_{k}$.

Coverage and logging (CL) is an effective check on XI in real-world applications because sparse or undocumented justifications may reduce the XI band, even if fidelity details or faithfulness appear to be sufficient.

The TI components, as shown in Table 1 assist in operationalizing the reproducibility and auditability throughout the secure software development life-cycle. Dataset versioning (DV) and model versioning (MV) are calculated from ad-hoc identifiers and archived releases. Pipeline logging (PL) is calculated by evaluating the status from partial logging to comprehensive, recent seed runs that include configurations, metrics, artifacts, and model lineage. Audit trail (AT) caters for traceability in the decision-making chain (input → model/version → explanation → decision → outcome). Reproducibility and replication (RR) quantify the reliability of reruns under a stated protocol and tolerances. Band thresholds encode concrete maturity milestones such as Digital Object Identifiers (DOIs) for data, image locks for environments, and tamper-proof logs which enables the auditors to conduct like-for-like reproducible re-runs given explicit configurations and environment.

The elements presented in Table 2 show the classification or score bands for each of the five risk tiers. Local fidelity (LF) components are quantified by the degree of agreement of surrogate models to the overall model behavior. Global stability or fidelity (GF) is measured by the consistency of the rankings of features or attributes across trains and splits. Faithfulness (FA) is classified by establishing the causality alignment that is assessed by perturbation curves, such as deletion/insertion AUC. Robustness (RS) is measured by the stability of the predictions and the explanations through benign perturbations and conducting stress tests. Coverage and logging (CL) is quantified by the percentage of decisions produced with explanations and context. Human comprehensibility (HC) is established using task-grounded probes such as time-to-insight, calibration, or decision quality for intended users. A good attribution metric without coverage or practical utility for the intended user will not yield a fairly high XI.

Risk tiers are classified by specifying threshold combinations (TI_min_, XI_min_). The eligibility in these cases is conjunctive because deployment is required to fulfill the condition of TI≥TI_min_ and XI≥XI_min_ with no active blockers. The assurance readiness score uses *t*_min_ = min(TI, XI) for assessment and communications, but is not a substitute for threshold checks. Any near-miss cases without blockers are flagged with WARN tags and come with suggested specific remediation derived from the lowest-scoring components. The violations of critical safety blockers, such as fairness gaps, PII leakage, and missing audit trails, will definitely result in BLOCK irrespective of index values.

Governance regulations and frameworks like EU, NIST, ISO 42001/23894, OECD/UNESCO, GDPR/ICO, FDA/IMDRF can fine-tune the threshold limits or corresponding weights but do not change the fundamental spirit of Tables 1, 2. For example, high-risk safety-critical domains may place greater emphasis on audit trails (AT) and reproduciblity/replication (RR) in traceability index (TI) or human-comprehensibility (HC) and coverage-loggin (CL) in explainability index (XI)„ consistent with risk-tier framing for high-risk use-cases in the EU AI Act and related domain-level trustworthy AI discussions (European Parliament and Council, 2024; Siddharth and Luo, 2025). Audit-as-Code is profile-driven and not bound to any single geography: it supports a baseline cross-jurisdiction profile grounded in broadly applicable standards like NIST, ISO 27001, ISO 42001, OECD/UNESCO principles, and applies jurisdictional or sectoral requirements as optional overlays or composed hybrid profiles when multiple regimes apply.

### 3.4 Policy, evidence schema, and gate configuration

**Figure 3:**
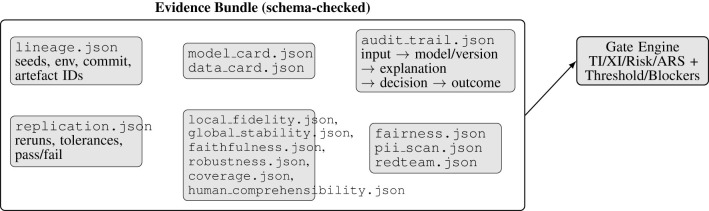
Evidence bundle structure. The gate consumes the same schema across runs to ensure consistent scoring and deterministic outcomes.

A versioned policy manages the deployment gate by formalizing the permissible risk levels of the asset, and categories for each level (TI_min_, XI_min_). The versioned policy defines the permissible evidence artifacts and schemas for required artifacts, the safety blockers that prevent deployment regardless of the scores, and the optional applicable governance requirements that help in narrowing down the explicit and specific thresholds that should be met without relaxing the critical safety blockers. These constraints on evidence required are both well-defined and machine-verifiable. The evidence bundles, such as shown in Table 4 with accompanying evidence schema in Figure 3 will be evaluated against JSON schemas, and each granularity of the assured readiness score must also be supported by parseable, linkable evidence, and not just a textual report. This structured representation is aligned with established argumentation approaches that emphasize on the explicit rationale and traceable justification structures for review, audit, and reuse (Aurisicchio et al., 2016; Siddharth et al., 2020).

**Table 4:** Required evidence artifacts (schema-checked).

**Key**	**File**	**Purpose/examples**
Lineage	lineage.json	*Seeds, container/python, git/commit, artefact IDs/hashes*
Model card	model_card.json	*Intended use, metrics, limits, monitoring hooks*
Data card	data_card.json	*Datasets, splits, preprocessing, provenance*
Audit trail	audit_trail.json	*input → model/version → explanation → decision → outcome chain*
Replication	replication.json	*seeded reruns; pass/fail vs. tolerances*
Explanation quality	local_fidelity. jsonglobal_stability. jsonfaithfulness.jsonrobustness.jsoncoverage.jsonhuman_ comprehensibility. json	*LF, GF, FA, RS, CL, HC sub-scores (0–1)*
Safety	fairness.jsonpii_scan.jsonredteam.json	*max_gap, PII hits, residual risk *R**
Risk	risk.json	*Composite risk and overlay fields (framework toggles)*

The normalized governance risk score used in ARS is bounded via *policy-defined, versioned bands* (Table 3) rather than assumed to be a universal scale from any single framework. Jurisdictional or sector overlays may adjust these band boundaries and/or tier thresholds, while preserving the same deterministic gate logic.

In our implementation, Risk ∈ [0, 1] is normalized and the policy defines the band thresholds used in Table 3, with optional overlays to tune mappings and thresholds.

Deployment decision outcomes are driven by a two-level policy. First, the gate determines *eligibility* of delegating *Pass, Warn, Block* by enforcing the tier-specific minimum thresholds on TI and XI, plus the non-overridable safety blockers. Second, the Assured Readiness Score (ARS) is used to select the *operational deployment mode* via score bands in Table 3. The low ARS bands restrict release to sandbox or internal-only environments. The intermediate bands permit only pilot or controlled rollouts and trigger mandatory review of the Fix-It remediation deltas before wider deployment. This makes the ARS actionable but does not replace the tier thresholds and blockers.

The lineage and model cards provide traceable identifiers for code, data, and models. This establishes the intended use and monitoring hooks. The audit trail encodes the input → model/version → explanation → decision → outcome chain. The deployment gate maintains a governance risk view for communication and triage. We summarize posture with an assured readiness score.


$$\begin{array}{l} \text{ARS}=\sqrt{\text{Risk}\cdot \min\left(\text{TI},\text{XI}\right)}, \end{array}$$ (4)

which emphasizes on the bottleneck dimensions in *t*_min_ = min(TI, XI) while preserving risk sensitivity. ARS classifications enable prioritization of review effort and operational decisions such as sandbox deployment with continuous audits, but eligibility for such deployment is decided strictly by meeting thresholds and the absence of critical safety blockers rather than dependent on ARS score alone. Table 3 provides a summary of nominal thresholds and corresponding decision outcomes. At each category, the gate provides a *PASS/WARN/BLOCK* decision and deltas for individually evaluated components against the evaluated thresholds alongside a short Fix-It plan that outlines the specific criteria it did not meet standard for, such as missing audit trail fields, insufficiently explained coverage of explanations.

### 3.5 Decision procedure, profiles, and continuous integration/continuous delivery (CI/CD) embedding

A deployment gate enforces applicable governance requirements by leveraging a versioned policy *P* (tiers, thresholds, blockers, weights, overlays) to a bind evidence schema *E* (Table 4). The readiness assurance is ensured by calculating the traceability index (TI) and explainability index (XI) derived from component sub-scores with fixed weights and maturity bands (Tables 1, 2). The deployability of the outcome is determined via tier-specific thresholds (TI_min_, XI_min_) and non-negotiable safety blockers (Table 3), ensuring that the readiness summaries cannot cover deficiencies along the limiting axis.

We enforce eligibility using separate tier-specific minima (*TI*_min_, *XI*_min_) and non-overridable safety blockers. These constraints are evaluated deterministically in the gate. ARS is not used to override any of these caps, but it provides a single operational summary for communication and prioritization. ARS enables ranking and triaging models within and across tiers to support deployment-mode guidance and highlights “near-miss” cases where the limiting dimension min(*TI, XI*) drives targeted remediation. The TI and XI thresholds determine the admissibility of the model while ARS summarizes readiness within the admissible space and supports consistent decision reporting.

**Figure 4:**
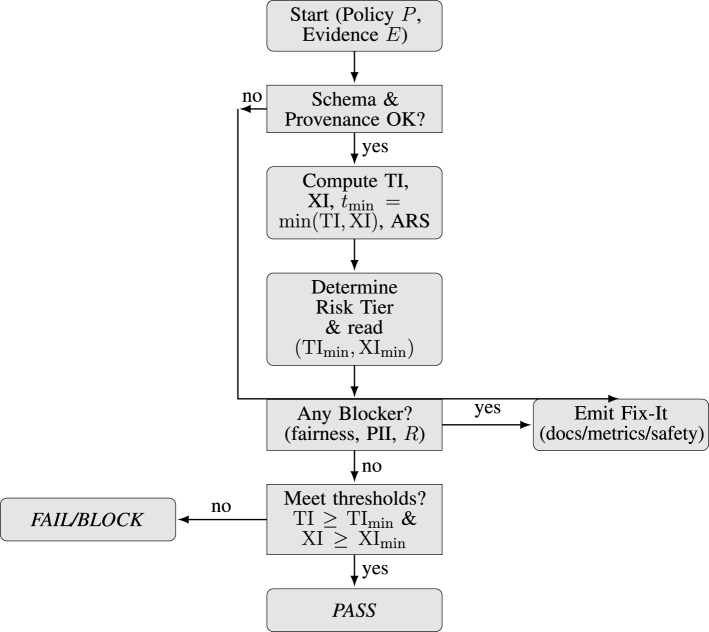
Gate decision flow: validate evidence, score TI/XI and ARS, apply tier thresholds and blockers, emit verdict and Fix-It.

Given policy *P* and evidence *E*, the gate executes the following deterministic sequence as illustrated in Figure 4:

1. Validate evidence: schema and provenance checks such as required keys, non-empty lineage, consistent hashes.
2. Score assurance: compute TI and XI from their component sub-weights as per Tables 1, 2.
3. Compose readiness: *t*_bottleneck_ = min(TI, XI) and $\text{ARS}=\sqrt{\text{Risk}\cdot t_{\text{bottleneck}}}$.
4. Determine tier: evaluate Risk and read (TI_min_, XI_min_) from *P* as in Table 3.
5. Check blockers: fairness gap, PII leakage, residual risk *R*; any hit ⇒ *BLOCK*.
6. Apply thresholds: if TI≥TI_min_ and XI≥XI_min_ with complete evidence, then *PASS*; otherwise **WARN**/*FAIL* per *P*.
7. Provide Fix-It suggestions: list missing artifacts, blocker remedies, and minimal deltas {ΔTI, ΔXI} that are needed to achieve minimal deployable thresholds.

This makes sure remediation to the limiting axis is kept separate while tier invariants are preserved.

Let Risk, TI, XI ∈ [0, 1] and $\text{ARS}=\sqrt{\text{Risk}\cdot \min\left(\text{TI},\text{XI}\right)}$.

If (TI′, XI′, Risk′)≥(TI, XI, Risk) elementwise, then ARS′≥ARS and thresholds once met remain met.

For fixed policy *P* and identical evidence *E* (same hashes/IDs), the Gate output is identical across executable cycles. Applicable governance regulations and frameworks might change the presentation of risk banding, but neither the thresholds (TI_min_, XI_min_) nor blocker predictions are affected by it. This means that any model that *PASS*es under base policy remains eligible independent of the applicable governance framework. The ∂ARS/∂TI = 0 whenever TI>XI (and symmetrically for XI), ensures the Fix-It objectives along the limiting axis.

The default (*strict*) profile applies the thresholds in Table 3 without relaxation. An optional *fallback* profile admits small Low/Moderate relaxations, e.g., 0.05 solely when there is no critical safety-blocker triggered and the documentary evidence in Table 4 is complete. Fallback reduces near-miss recalculations while preserving blocker invariants and prohibiting relaxations in higher-risk tiers.

### 3.6 Threat model and mitigations

We consider data, model, pipeline, and interface threats following MITRE Corporation (2024) and OWASP Foundation (2024). The deployment gate enforces evidence and critical safety-blockers that correspond to common risks, such as:

- Data provenance and tamper: lineage hashes, versioned datasets (lineage.json, DV/MV) ⇒ traceable rollback and audit.
- Model supply chain: hashed and verified artifacts, and build attestations (SLSA-style fields) ⇒ verifiable integrity.
- Prompt interaction risks: red-team residual risk *R* and PII scanning ⇒ *blockers* if *R*>0.25 or high-severity PII.
- Reasonable and sufficient explanations: stability/faithfulness/robustness evidence (XI) ⇒ thresholds that prevent brittle deployments.

All policies, schemas, and CI examples are available at (Muhammad 2025).

## 4 Evaluation

We evaluate whether *Audit-as-Code* translates principles of governance into operational, actionable, and reproducible technical outcomes across model settings and jurisdictions. Every scenario yields two categories of outcomes:

1. Readiness (operational) via the Assured Readiness Score $\text{ARS}=\sqrt{\text{risk}\cdot \min\left(\text{TI},\text{XI}\right)}$, mapped to deployment bands (sandbox → deploy with audits);
2. Minimum assurance (safety) via a *tier gate* enforcing tier thresholds (TI_min_, XI_min_), required artifacts, and blockers (fairness/PII/residual risk).

Reporting both of these outcomes prevents category errors. A strong ARS cannot mask missing lineage or weak explainability under a high-risk tier.

### 4.1 Corpora, policies, overlays, and scoring setup

We assess the performance of Audit-as-code on two separate corpora: The synthetic corpus and the Real-World models. The Synthetic corpus consists of 1,000 scenarios that are synthesized across a variety of modalities, such as LLM, CV, tabular, and time series, across seven tiers of CORTEX (Muhammad et al., 2025) tiers. Each of the 1,000 scenarios come with a full evidence bundle including lineage, model cards, data cards and audit trails. The explanation quality metrics of all explainability index (XI) components, LF, GF, FA, RS, CL, and HC, are also evaluated for all these 1,000 scenarios. The corpus also integrates principles of fairness, PII sensitivity, red-teaming metrics, and risk.json).

The Real-world model corpus consists of five models, such as Adult XGB, Toxic DistilBERT, ResNet-50/CIFAR-10, Electricity/TFT, and Llama-3. To make the real-world evaluation setup explicit and comparable across modalities, Table 5 summarizes each real-world system by (i) modality and task context, (ii) the primary application domain represented by the benchmark, and (iii) the scale of the underlying dataset and model/configuration used in our evaluation.

**Table 5:** Real-world model corpus used in evaluation: domains and scale.

**System**	**Modality**	**Task & domain**	**Scale/size (data + model)**
Adult XGB	Tabular	Binary classification (income prediction); representative socio-economic decision setting often used in governance/fairness discussions	Data: Adult/Census Income (UCI), 48,842 instances; common split 32,561 train/16,281 test (UCI Machine Learning Repository, 2024). Model: XGBoost tree ensemble (Chen and Guestrin, 2016).
Toxic DistilBERT	Text	Multi-label toxicity detection for content moderation/platform safety (user-generated content)	Data: Jigsaw Toxic Comment Classification Challenge, 159,571 train/153,164 test comments (Kaggle, 2018). Model: DistilBERT-family toxicity classifier (model card reference) (Unitary Team, 2021).
ResNet-50/CIFAR-10	Image	10-class object recognition benchmark; representative computer vision classification workload	Data: CIFAR-10, 60,000 images (32 × 32), 50,000 train/10,000 test (Krizhevsky, 2009). Model: ResNet-50 reference implementation (PyTorch Vision Team, 2025).
Electricity/TFT	Time-series	Multi-horizon forecasting for electricity consumption (utilities/energy demand management)	Data: ElectricityLoadDiagrams20112014 (UCI): 370 series/clients; 140,256 time steps per series (15-min sampling) (UCI Machine Learning Repository, 2015). Model: TemporalFusionTransformer reference implementation (Beitner and contributors, 2025).
Llama-3 (local)	Text (LLM)	General-purpose instruction-following/assistant-style generation (cross-domain)	Model: Meta-Llama-3-8B (8B parameters), local inference (Meta AI, 2024). Evaluation set: 200 prompt-based test cases spanning policy profiles (governance tiers/overlays).

Each system is summarized by modality/task, major application domain, dataset scale, and model scale/configuration.

While specific jurisdictional and governance requirements from the EU AI Act, the NIST AI RMF, ISO/IEC 42001/23894, OECD/UNESCO, GDPR/ICO, and FDA/IMDRF will change CORTEX modifiers (*C, G, T, E, R*) (Muhammad et al., 2025). And consequently lead to changes in per-framework risk_*f*_ and ARS_*f*_. The Gate minimums and critical safety-blockers retain their governance-framework-invariance. The scoring criteria follows earlier definitions such as of calculating CORTEX risk via a utility transform *U*(*L, I*) = 1 − exp(−*kLI*) with overlays; ARS produces continuous, weighted TI/XI from banded components; the bottleneck is *t*_bottleneck_ = min(TI, XI) and $\text{ARS}=\sqrt{\text{risk}\cdot t_{\text{bottleneck}}}$. A scenario passes the deployment Gate if and only if all required artifacts are present, no critical safety-blocker is triggered, and TI≥TI_min_, XI≥XI_min_ for its tier. Table 6 summarizes strict-policy outcomes: 409 *PASS* vs. 591 *FAIL*; mean TI/XI/ARS = 0.93/0.68/0.64.

**Table 6:** Corpus summary under strict policy (1,000 scenarios).

**Metric**	**Value**
Scenarios (total)	1,000
gate *PASS*	409
gate *FAIL*	591
mean *TI/XI/ARS*	0.93/0.68/0.64

Under the strict profile, the deployment gate passed 409 of 1,000 scenarios and failed 591. Based on the data provided (Table 6), the mean TI is significantly higher than the mean XI (0.93 vs. 0.68). This difference illustrates that the limiting axis for eligibility is the explainability index (XI) as opposed to the traceability index (TI). The ARS score of 0.64 suggests a bottleneck at the base of the approach to eligibility, i.e., XI index. This demonstrates that we can yield the largest marginal improvement in eligibility by improving the fidelity, stability, and faithfulness of explanations under the strict policy.

**Figure 5:**
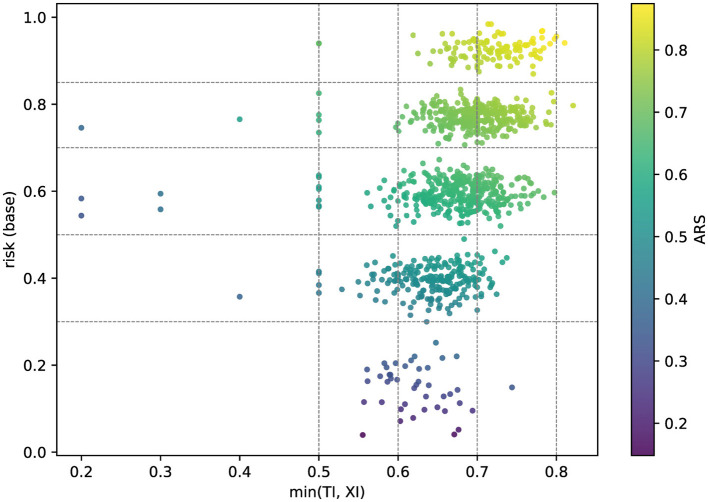
Quadrant over 1,000 scenarios (strict): *y* = risk; *x* = *t*_bottleneck_; color = ARS; horizontal tier lines; vertical TI/XI guidance.

Figure 5 plots *y* = risk vs. *x* = *t*_bottleneck_ = min(TI, XI) with tier lines and TI/XI guidance. Dense bands just to the right of vertical guides indicate near-misses—typically fixed by small XI uplifts or documentary completion.

**Figure 6:**
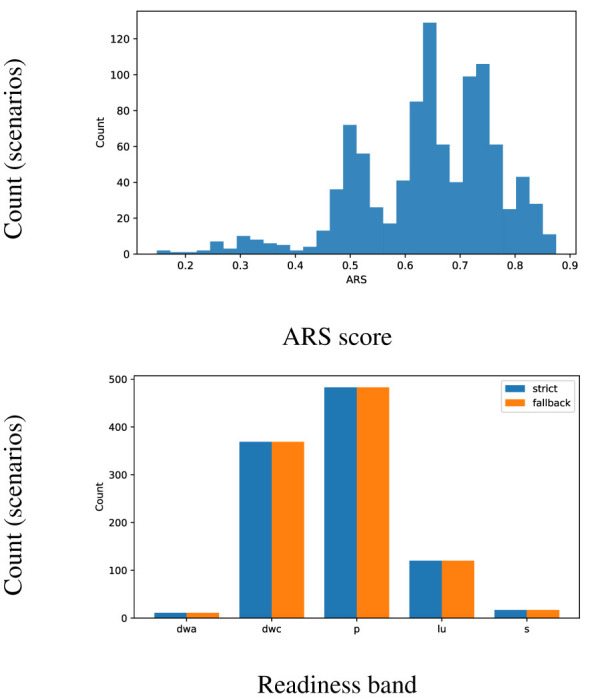
Readiness overview: ARS histogram (strict) and band composition (strict vs. fallback).

Figure 6 shows the ARS histogram (strict) and band composition (strict vs. fallback). Most scenarios are classified in the middle of the readiness categories (pilot/controls). Fallback module changes near-miss non-blocked cases with an increase in Gate *PASS* rate, while bands themselves remain governed by ARS.

Within the parameters of the strict profile, the majority of situations lie in the mid-readiness categories as shown in Table 7. We can see that *pilot* represents 483 cases (48.3%) and *deploy with controls* represents 369 cases (36.9%). Only a small tail exists under the *deploy with audits* readiness band with 11 cases (1.1%), and only a few lie in *sandbox* deployment mode with 17 cases (1.7%). Overall, this distribution in deployment modes demonstrates near-universal eligibility, primarily restricted by explanation quality at the current appropriate level, with only a small number of situations outranked in the top assurance band without monitoring.

**Table 7:** ARS bands under strict policy.

**Band**	**Count**	**Share**
deploy_with_audits (≥0.85)	11	1.1%
deploy_with_controls	369	36.9%
pilot	483	48.3%
limited_use	120	12.0%
sandbox	17	1.7%

Table 8 further subdivides strict fail cases into categories such as missing evidence, critical safety-blockers such as fairness, PII, *R*) and threshold deficit only with no blockers, but TI or XI under threshold. This sub-division or sub-categorization justifies the Fix-It playbook, i.e., by adding missing required evidence or documentation, by fixing critical safety-blockers, or by nudging near-threshold scenarios, the failure outcomes can be flipped. Table 8 shows the most common reasons associated with gate failures tabulated as a share of the total corpus. The leading contributor is the missing required evidence, with 267 cases. Among critical safety policy blockers, the fairness gap exceeding 0.10 occurs in 166 cases, high residual risk *R*>0.25 occurs in 95 cases, and high sensitivity PII leakage occurs in 56 cases. An additional 125 cases reflect the threshold deficits, i.e., where there are no missing artifacts or critical safety-blockers, but TI or XI is below the applicable threshold. Overall, this data in Table 8 shows that completeness of documentation is the most common barrier, while critical safety-blocker breaches are less common, but have a severe impact if they occur.

**Table 8:** Principal failure causes under strict policy (shares are of the full corpus).

**Reason class**	**Count**	**Share**
Missing required evidence	267	26.7%
High residual risk (*R*>0.25)	95	9.5%
PII leakage (high severity)	56	5.6%
Fairness gap (> 0.10)	166	16.6%
Threshold deficit only (no missing/blockers; TI or XI below tier threshold)	125	12.5%

For critical safety-blockers related to fairness assurance, the evaluation of the Toxic DistilBERT model shows a maximum gap of 0.12. When we apply post-processing, the gap is reduced to 0.08, changing *FAIL*→*PASS*. For PII/*R* blockers, Llama-3 initially failed due to high-severity leakage critical safety-blocker and had *R* = 0.30. This enables us to implement input/output filters and strengthen guardrails, reducing PII to minimal and *R* to 0.20, changing *FAIL*→*PASS*.

Under the relaxed fallback profile where thresholds for Low and Moderate bands is reduced by approximately 0.05 with critical safety-blockers unchanged, the *PASS* rate of the gate increases from 40.9 to 49.7%. This comes with a change of Δ= 8.8 percentage points while the median ARS remains 0.652 (see Table 9). The newly passed eighty-eight scenarios comprise mostly of the near-miss scenarios that were previously failing on defined thresholds only. The failure cases related to fairness gaps, PII leakage, or *R*>0.25 remain blocked because the fallback profile does not relax critical safety-blockers or require compulsory evidence or documentation.

**Table 9:** Strict → fallback sensitivity.

**Metric**	**Value**
Gate PASS rate	40.9% → 49.7% (Δ +8.8pp)
Median ARS	0.652 → 0.652 (unchanged)
New PASS	88 (primarily prior threshold deficits)

### 4.2 Real-world audits

As per the default policy with strict thresholds and critical safety-blockers, three of the real-world models are classified in moderate tiers of deployments as they meet the tier thresholds without triggering blockers. And two of the real-world models are classified as High-tier, and they fail due to safety conditions. Table 10 reports continuous TI, XI, residual risk *R*, composite ARS, readiness bands, and deployment gate decisions alongside a compact evidence bundle. The Moderate tier models tabular, time-series, and vision achieve TI ∈ [0.88, 0.95] and XI ∈ [0.62, 0.71] and therefore *PASS*. The High-tier NLP model fails on a fairness gap exceeding 0.10, and the local LLM fails on high-severity PII leakage with *R*>0.25; both these cases illustrate that critical safety-blockers dominate the decision regardless of strong TI or ARS.

**Table 10:** Real-world audits (strict, base policy): continuous TI/XI and gate.

**Model**	**Tier**	**Evidence**	**TI**	**XI**	**R**	**ARS**	**Band**	**Gate**	**Issue/Fix-It**
Adult XGB (tabular)	Moderate	L, C, R, A, X	0.92	0.71	0.08	0.65	p	PASS	–
Electricity/TFT (time-series)	Moderate	L, C, R, A, X	0.88	0.69	0.10	0.62	p	PASS	–
ResNet-50/CIFAR-10 (vision)	Moderate	L, C, R, A, X	0.95	0.62	0.00	0.61	p	PASS	Low HC ⇒ add summaries
Toxic DistilBERT (NLP)	High	L, C, R, A, X, F	0.90	0.70	0.20	0.73	dwc	FAIL	Fairness >0.10 ⇒ post-process
Llama-3 (LLM)	High	L, C, R, A, X, RT, PII	0.96	0.67	0.30	0.74	dwc	FAIL	PII; *R*>0.25 ⇒ filters/guardrails

Evidence codes: L, lineage; C, cards; R, replication; A, audit_trail; X, XAI; F, fairness; RT, red-team; PII, PII scan. Band codes: p, pilot; dwc, deploy_with_controls; (lu=limited_use; dwa=deploy_with_audits; s, sandbox).

We observe that the models evaluated against the EU-aligned profile yield the highest failure rate in our comparative audit runs. The model evaluated against the EU AI Act is bounded by a broader set of mandatory evidence requirements and needs to adhere to stricter non-overridable safety conditions. In practice, this increases the probability that a real-world system triggers at least one blocking condition like residual safety findings or missing or insufficient evidence for required controls, even when other governance profiles would permit a restricted rollout. Therefore, the higher failure rate when evaluating against the EU AI Act reflects the profile's stronger emphasis on precautionary gating under uncertainty and incomplete evidence rather than a change in the underlying model performance.

To ensure a portable cross-regulation implementation of the models in a way that does not compromise constraint thresholds, we enable light documentation/safety-style presets that are compatible with frameworks such as EU/ICO, data protection impact assessment, and records of processing as per FDA/IMDRF, verification/validation, and change control as per OECD/UNESCO. As summarized in Table 11, these add-ons enable achieving realistic pre-fix gaps. The time-series model is flagged for missing Data Protection Impact Assessment (DPIA) documentation/records as per EU/ICO requirements. The vision model is flagged for missing human-centered transparency notes. Whereas the NLP and LLM models fail uniformly on violations of fairness and PII/*R*, respectively. More importantly, the underlying TI/XI thresholds remain unchanged. And these presets only activate evidence checks and safety hooks that many jurisdictions already expect.

**Table 11:** Framework Gate outcomes *before* remediation (strict policy + minimal framework Gate presets).

**Model**	**EU**	**NIST**	**ISO 42001**	**OECD/ UNESCO**	**ISO 23894**	**ICO/GDPR**	**FDA/ IMDRF**
Adult XGB	PASS	PASS	PASS	PASS	PASS	PASS	PASS
Electricity/TFT	FAIL (DPIA)	PASS	PASS	PASS	PASS	FAIL (audit)	PASS
ResNet-50/CIFAR-10	FAIL (HC)	PASS	PASS	FAIL (HC)	PASS	FAIL (HC)	PASS
Toxic DistilBERT	FAIL (fair)	FAIL (fair)	FAIL (fair)	FAIL (fair)	FAIL (fair)	FAIL (fair)	FAIL (fair)
Llama-3 (local)	FAIL (PII,*R*)	FAIL (PII,*R*)	FAIL (PII,*R*)	FAIL (PII,*R*)	FAIL (PII,*R*)	FAIL (PII)	FAIL (PII,*R*)

DPIA, impact assessment/record; audit, records of processing; HC, human-centric transparency note; fair, fairness gap >0.10; PII, missing PII filters; *R*, residual risk >0.25.

#### 4.2.1 Cross-framework gate (pre/post)

**Figure 7:**
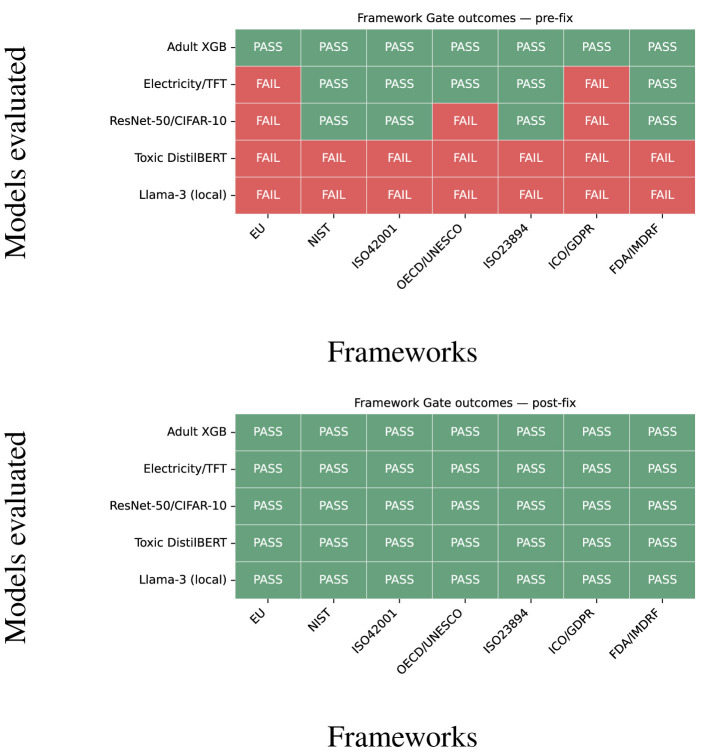
Framework gate outcomes. **(Left)** Pre-fix with minimal presets. **(Right)** Post-fix after minimal remediation. Gate thresholds never change; only documentary/safety checks differ.

To fix and address the specific failures, we implement the least restrictive appropriate fixes while maintaining the existing policy thresholds. For the NLP model, we bring the fairness gap to ≤ 0.10 through post-processing by fixing the ground truth. For the LLM lags, we enable PII filtering and guardrails and reduce the *R* ≤ 0.25 residual risk. For models without documentation, we propose specific DPIA records and a brief human-centric actionable note. Figure 7 shows the difference between mixed outcomes pre-fix (on the left) and post-fix scenarios to *PASS* across the highlighted frameworks (on the right). It also shows that the specific fixes decrease the compliance deltas without impacting the assurance readiness score.

#### 4.2.2 Minimal fix-it remedies

**Figure 8:**
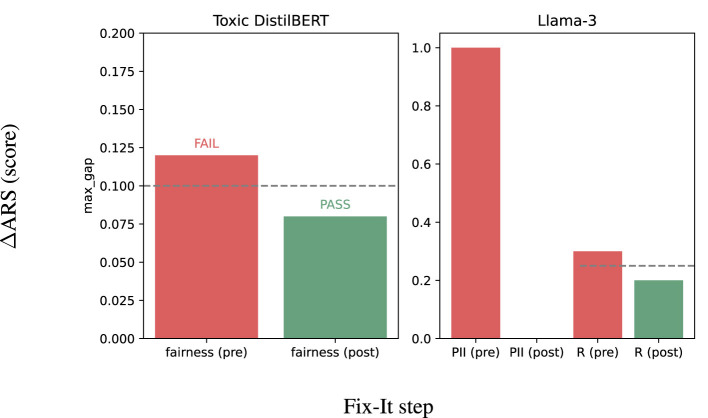
Minimal fix-it: toxic (fairness ≤ 0.10) and Llama-3 (PII filters + *R* ≤ 0.25) flip FAIL → PASS; readiness bands remain consistent with High-tier controls.

The magnitude of changes and fixes is compact and is in line with the policies. The changes are summarized visually in Figure 8, and provide the decisive adjustments, for example, adjusting fairness from 0.12 → 0.08 for the NLP model, and PII 1 → 0 referenced to *R*:0.30 → 0.20 for the LLM. In all these cases, the changes assist in reversing the verdict from **FAIL** to *PASS* while the readiness bands remain within High-tier deploy_with_controls. This consistency suggests that the limiting factor was a blocker rather than an underlying shortfall in TI or XI.

**Figure 9:**
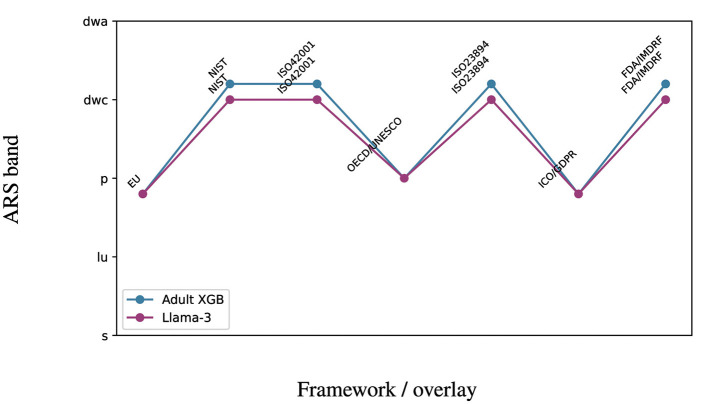
Overlay readiness by framework (slopegraph): adult vs. Llama-3. Jurisdictional overlays move ARS bands; Gate thresholds remain invariant.

Jurisdictional overlays suggest readiness levels, but they do not modify spatial thresholds or blockers. Figure 9 demonstrates band classifications across frameworks for a prototype AdultXGB that is classified in medium-tier vs. the post-fix High-tier LLM. The relative stability of bands post-remediation indicates that if blockers are resolved and artifacts are required, it can lead to minimal variation across frameworks. The deployability of the model still is dependent on its classification within tier thresholds and protection.

### 4.3 Comparative baselines

We also evaluate the deployment gate against three reference points reflecting good governance practices:

(i) *Documentation-only (Docs)*: in this benchmark scenario, the model *PASS*es if required artifacts and evidence as suggested in Table 4 are present. And is not evaluated for quantitative TI/XI checks or safety probes.
(ii) *Score-only (Scores)*: in this benchmark scenario, the model *PASS*es if the weighted TI/XI meet tier thresholds as classified in Table 3, and ignores the safety blockers altogether.
(iii) *Framework checklists (Checklists)*: in this benchmark scenario, the model *PASS*es if minimal framework documentation, as in Section 4.4 is present, and is not evaluated on quantitative thresholds.

The final decision is evaluated against the gate as reference using accuracy, precision, recall, *F*_1_, Cohen's κ, and a false-pass rate with respect to safety ground truth (cases a baseline passes while any blocker is present: fairness gap ≥0.10, PII leakage, or residual risk *R*≥0.25).

Table 12 compares three common reference strategies to the proposed deployment gate. Documentation-only reference strategy has limitations as it achieves an accuracy of 0.590 and a precision of 0.520 with a very high false-pass rate of 0.410 against ground truth for safety. This means approximately 40% of cases failing the safety test would be permitted into the system if checking only for the presence of the artefact or evidence was used as the criterion for deployment. Framework checklists strategy performs about the same with the accuracy of 0.640 and precision of 0.550. This still allowed 0.380 of cases that failed against safety ground truth, providing other indications of risk in the checklist. Although these strategies might provide some assurance regarding the safety and decency of the documentation used. The similarity in false passes yielding considerable scores demonstrates the risk of relying on documentation or checklists alone. Heuristic score criteria with no reference to blockers improved the metrics with an accuracy of 0.780 and a precision of 0.720, but still allowed 0.190 of safety-failing cases. This primarily occurs because it allows room for fairness, a PII leakage, and residual risk violations. As intended while proposing, the deployed gate mechanism integrated equals zero false passes, meaning blockers are non-overridable, and thresholds are an explicit statement of minimum requirements to comply with quantitative assurance. This net effect is a criteria that is stricter but explainable. This ensures that models may increase the score for TI or XI to comply with a threshold, but no changes within the documentation would be enough to override the critical safety-blockers.

**Table 12:** Comparative baselines vs. the gate (strict policy; *n* = 1, 000).

**Method**	**Acc**.	**Prec**.	**Rec**.	**F_1_**	**κ**	**False-pass (safety)**
Gate (proposed)	1.000	1.000	1.000	1.000	1.000	0.000
Docs (presence only)	0.590	0.520	0.890	0.660	0.180	0.410
Scores (no blockers)	0.780	0.720	0.840	0.770	0.550	0.190
Checklists (framework)	0.640	0.550	0.880	0.680	0.270	0.380

The gate is the reference.

### 4.4 Ablations, robustness, and CI-like overhead (1,000 scenarios)

We conduct a policy sanity check on five of the models to test the strict and fallback thresholds. The fallback thresholds relax the low-tier thresholds by about 0.05, but never waive required documentation or critical safety blocks. As observed in Table 14, there are no changes in outcomes for all five models. All three models classified in the moderate tier: AdultXGG, Electricity/TFT, and ResNet -50 passed in strict thresholds. And they remained being in *PASS* classification. The models classified in High-tier models: Toxic DistilBERT, Llama -3 remain in *FAIL* category until their safety blocks are remediated, such as fairness gap (0.10); PII leakage, and (*R*>0.25). This check verifies that fallback serves as a guardrail for cases of near-miss, rather than being a waiver of documentation or safety conditions.

We also examine decision stability, evidence ablations, and runtime environment CI-like simulation at the corpus scale. The Table 13 summarizes the following three considerations. First, gate decisions demonstrate stability against moderate small weight or noise perturbations with stability of 94.0%, and with modest changes in summary scores (mean ΔTI = 0.006, ΔXI = 0.021, ΔARS = 0.010). Second, ablative features to core artifacts result in forced failures. This suppresses the limiting axis below thresholds, and removing the lineage (TI → 0) yields *FAIL* in 96.0. The degrading stability starting at 0.06 results in XI thresholds below 82%. Furthermore, increasing residual risk *R* to 0.40 results in blocking all cases. Third, the CI-simulated overhead is negligible at per-scenario median and 90th percentile latencies are effectively zero when collectors are cached. The synthetic corpus scenario batch completes in 4.5 s on the test host.

**Table 13:** Robustness, ablations, and CI-like latency (1,000 scenarios).

**Metric**	**Value**
Gate stability (perturbations)	94.0%
Mean ΔTI/ΔXI/ΔARS	0.006/0.021/0.010
Remove lineage (TI → 0) ⇒ FAIL	96.0%
Drop XI stability (−0.06) ⇒ FAIL	82.0%
Raise residual risk (*R* = 0.4) ⇒ blocked	100.0%
Per-scenario latency (p50/p90)	0.0s/0.0s
End-to-end batch time	4.5s

The Table 14 indicates that the Fallback relaxes a low-tier threshold by approximately 0.05, but does not concede documented evidence expectations and critical safety-blockers. Because thresholds and blockers are not affected, the framework presets illustrate minimal requirements needed for documentation and safety adjustments that are accomplish-able. The Table 15 lists the minimal changes as short DPIA or impact records and processing or audit notes as per the EU AI Act and ICO/GDPR. It also shows basic V&V and change-control paperwork as per FDA/IMDRF. The governance/risk artifacts required as per NIST RMF, ISO/IEC 42001, and ISO/IEC 23894. And a short human-centred transparency note as per OECD/UNESCO. In all practicality, all of these requirements apply asymmetrically to all the models. The time-series system generally is missing documentation for DPIA/audit paperwork. While the vision system will benefit from an HC note to reviewers. On the other hand, the local LLM model requires filtering and guardrails for PII to have *R* ≤ 0.25. None of these changes would affect the TI/XI thresholds. They simply reflect expectations of jurisdictions that many organizations already encounter.

**Table 14:** Gate under strict vs. fallback (real-world).

**Model**	**Gate (strict)**	**Gate (fallback)**	**Reason**
Adult XGB	PASS	PASS	thresholds met; no blockers
Electricity/TFT	PASS	PASS	thresholds met; EU/ICO may still expect docs (DPIA/audit record)
ResNet-50/CIFAR-10	PASS	PASS	thresholds met; HC transparency note expected by OECD/EU/ICO reviewers
Toxic DistilBERT	FAIL	FAIL	fairness blocker (>0.10) dominates; fallback cannot override
Llama-3 (local)	FAIL	FAIL	PII and *R*>0.25 blockers dominate; fallback cannot override

**Table 15:** Framework-specific minimal requirements and affected models (pre- and post-fix).

**Framework**	**Minimal requirement**	**Affected (pre-fix)**	**Fix-It to PASS (post-fix)**
EU AI Act	DPIA/impact record; PII filters where applicable	Electricity (docs); Llama-3 (PII)	Add DPIA; add PII filters; Llama-3 also harden guardrails (*R* ≤ 0.25)
ICO/GDPR	Records of auditability; PII filters	Electricity (docs); Llama-3 (PII)	Add audit record; add PII filters; Llama-3: *R* ≤ 0.25
FDA/IMDRF	V&V and change-control artifacts	none (all have minimal V&V)	Adult/Electricity/ResNet bands ↑ with stronger V&V
NIST RMF	RMF governance evidence (MAP/MEASURE/MANAGE/GOVERN)	none (all OK)	Bands steady or ↑ with MLOps evidence
ISO/IEC 42001	AIMS policies/roles/audit log extensions	none (all OK)	Bands steady or ↑ with AIMS artifacts
ISO/IEC 23894	Risk-treatment/monitoring record	none (all OK)	Bands steady or ↑ with treatment log
OECD/UNESCO	Human-centric transparency/explanation note	ResNet-50 (HC note)	Add HC note (one paragraph in model card appendix)

The Table 15 illustrates that DPIA or audit records are normally presented as documentation gaps for Moderate-tier operational procedures. The PII leakage guardrails and *R* ≤ 0.25 represent blocking issues for LLMs. And a short HC note effectively serves to substantially resolve disputes associated with review based upon a vision model. All these observations do not modify TI/XI thresholds as they simply provide continuity and provide correlation between Gate checks and documentation required across governance regulations and frameworks. We also consider the nature of governance frameworks and how they interact and change readiness postures while the deployment gate decisions remain constant. The Table 16 shows the changes in band classification against the base profile. In all the cases, these changes were minute and model dependent as in the cases of pilot ↔ deploywithcontrols.

**Table 16:** Per-framework overlays: readiness bands (relative to base).

**Model**	**EU**	**NIST**	**ISO42001**	**OECD/UNESCO**	**ISO23894**	**ICO/GDPR**	**FDA/IMDRF**
Adult XGB	p↓	dwc↑	dwc↑	p↔	dwc↑	p↓	dwc↑
Electricity/TFT	p↓	p↔	dwc↑	p↔	dwc↑	p↓	dwc↑
ResNet-50/CIFAR-10	p↓	p↔	p↔	p↔	p↔	p↓	p↔
Toxic DistilBERT	p↓	dwc↔	dwc↔	p↔	dwc↔	p↓	dwc↔
Llama-3 (local)	p↓	dwc↔	dwc↔	p↔	dwc↔	p↓	dwc↔

Band codes: p, pilot; dwc, deploy_with_controls. Arrows ↑/↓ show small up/down shifts in ARS band due to overlay risk.

### 4.5 Uncertainty and sensitivity

We apply uncertainty to our corpus statistics and for proportions such as Gate *PASS*, band classifications, and we report Wilson 95 confidence intervals. For this purpose, we use means to report nonparametric bootstrap 95 intervals over ten thousand resamples as the basis for computing confidence intervals. To conduct sensitivity analyses, we vary traceability index (TI) and explainability index (XI) weights by ±0.05 via simplex re-normalized and inject *N*(0, 0.02^2^) noise into component sub-scores that recomputes Gate outcomes and ARS classifications. We compute the Wilson interval as follows: for a proportion $\hat{p}=k/n$ and *z* = 1.96,


$$\text{C}{\text{I}}_{\text{Wilson}}=\frac{\hat{p}+\frac{z^{2}}{2n}\pm z\sqrt{\frac{\hat{p}\left(1-\hat{p}\right)}{n}+\frac{z^{2}}{4n^{2}}}}{1+\frac{z^{2}}{n}}.$$

We then resample scenarios with replacement to compute bootstraps for means over ten thousand draws, recompute the statistic, and then report the 2.5 and 97.5 percentiles.

In Table 17, summary statistics show sampling uncertainty around the corpus summaries. The Wilson interval for the *PASS* proportion was [0.379, 0.440], indicating a width of 0.061 around the point estimate 0.409. This width and point estimation suggests that even accounting for binomial variability, the observed pass rate is substantially below one-half. The mean TI was tightly concentrated within [0.92, 0.94], indicating strong traceability. The XI mean was within [0.66, 0.70], which shows that in the vast majority of the scenarios, explainability is the limiting axis. The mean ARS score was between [0.62, 0.66] and was consistent with the band distributions reported in the main results. The Wilson interval for the *pilot* was [0.452, 0.514], meaning roughly most of all cases fall into the pilot readiness category. The Sensitivity to noise and decision weight had little impact on the decision. The decision changes remain at 0.940, and the average observed absolute changes were ΔTI = 0.006, ΔXI = 0.021, and ΔARS = 0.010, numerically and materially below, the tier threshold decision gaps that have been included in policy, which are typically 0.05. In summary, both the statistics and the gate decisions appear consistent and stable when tested with reasonable perturbations.

**Table 17:** Uncertainty (95 confidence intervals) under strict policy (*n* = 1, 000).

**Metric**	**Point estimate**	**95 CI**
Gate PASS proportion	0.409	[0.379,0.440]
Mean TI	0.93	[0.92,0.94]
Mean XI	0.68	[0.66,0.70]
Mean ARS	0.64	[0.62,0.66]
Top band share (pilot)	0.483	[0.452,0.514]

We emphasize comparative baselines, ablation-style checks, and uncertainty to strengthen the empirical analysis. Specifically, we compare the proposed gate decisions against simpler alternatives like checklist or documentation-only approaches, and present the differences of weak gating using false-pass behaviour relative to blocker-triggering cases as shown in Table 12. We also examine how policy choices affect outcomes by contrasting strict vs. fallback thresholding Section 4.4, ensuring that near-miss cases do not weaken blocker guarantees. Finally, we evaluate uncertainty by Wilson confidence intervals and bootstrap resampling, and perform sensitivity tests by perturbing weights and injecting controlled noise as referred in Table 17, showing that the qualitative conclusions are stable and not artifacts of narrow parameter settings.

## 5 Discussion

This study aimed to map the governance requirements from textual details into quantifiable checks with deterministic outcomes. In this section we comment on the results and link them to design decisions in Methods, and summarize what actions engineering or planning teams relevant to AI model designing and deployment should consider. When reviewing documentation such as models or data cards, the provenance documentation helps to establish situational context for the reviewers and auditors, but it does not ensure absolute sufficiency, nor is it a final decision-making artefact. The deployment gates bridge this gap by mapping evidence to maturity bands (Tables 1, 2). This helps in enforcing tier thresholds (Table 3), and issuing framework-agnostic critical safety-blockers. The bottleneck *t*_bottleneck_ = min(TI, XI) prevents obfuscating the weak axes. Through evaluating synthetic and real cases, we can have an asymmetric assurance, such as solid traceability but weak explanation coverage/quality. It produces a moderate ARS but does not breach thresholds, a desired behavior for safety monitoring. In contrast, specific improvements along the limiting axis change the outcomes in a favorably predictable way. The ARS score remains constant for communication/triage, and eligibility is very specifically threshold/blocker-based.

**Figure 10:**
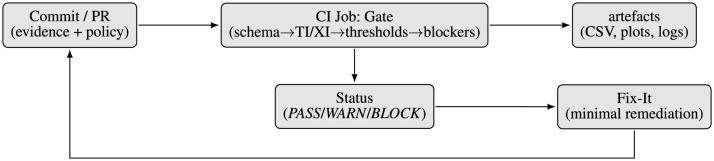
Continuous integration/continuous delivery (CI/CD) integration: deterministic gate runs on each PR, emitting artifacts, status, and Fix-It; re-runs are reproducible (seeds, container, commit, policy version).

Because the same engine runs locally and in CI (Figure 10), decisions are reproducible and verifiable when policy, evidence, or code change. Schema checks, artefact hashing, and explicit audit-trail links deter unverified modifications. Table 18 shows the minimal fix-it remediations required for some of the observed reasons for failure during this research, outlining the symptoms of failure, and minimal remediations needed in each case to fix the respective failures.

**Table 18:** Fix-It playbook. Smallest remediation that deterministically flips outcomes when possible.

**Failure class**	**Symptoms/gate outcome**	**Minimal remediation**
Missing documentary artifacts	EU/ICO overlay withholds *PASS* pending records; reviewer cannot trace accountability (*FAIL*)	Attach records of processing/DPIA; add a brief auditability note in the model card; re-run gate (no metric changes).
TI threshold deficit (near-miss)	TI < TI_min_ by < 0.05; weak PL/AT links; incomplete lineage (*FAIL*)	Enable training+inference logging; link artifacts and run params; add environment lockfile and seed; re-score.
XI threshold deficit (near-miss)	XI < XI_min_ by < 0.05; low coverage or weak HC (*FAIL*)	Increase explanation coverage to threshold; add HC transparency note/appendix; retain exemplar explanations; re-score.
Fairness blocker	Max protected-attribute gap >0.10 (*BLOCK*)	Apply post-processing (reweighting, thresholding, equalized odds) to achieve ≤ 0.10; re-probe and re-score.
PII blocker	Sensitive entities in inputs/outputs (*BLOCK*)	Enable input and output PII filters; sanitize logs; add validation tests; re-probe and re-score.
Residual-risk blocker	Red-team residual risk *R*>0.25 (*BLOCK*)	Harden guardrails (jailbreak-pattern filters, content-safety classifiers, tool-use limits) to achieve *R* ≤ 0.25; re-probe.

Smallest remediation that deterministically flips outcomes when possible.

### 5.1 Limitations

The proposed framework operationalizes traceability index (TI) and explainability index (XI) as evidence-driven and reproducible indices that contribute significantly to an assurance posture. By design, this framework focuses on quantifiable artifacts and evidence (Figure 3), and tier thresholds (Figure 3). However, the framework design ensures that there is no inherent exhaustion of the qualitative aspects of responsible deployment, such as organizational culture, stakeholder trust, or social norms of a domain. Hence, the flexibility is there to tune the modifiers, and thresholds can be interpreted as necessary rather than sufficient conditions for release. The results are dependent on the quality and completeness of the evidence submitted. More noisy or incomplete documentation can cause schema validation to fail and cause sub-scores to fall similarly. This design strategy makes sure that well-governed systems, even without enough documentation, will underperform. The framework has room for some elements of expert judgment, for example, judgment of human comprehensibility in XI, which is consistent with established practice when studying explainability.

### 5.2 Future work

Weights and thresholds defined in this framework are policy parameters and not universally viable constants. The default weights we have used are reasonable as starting points, but regulated or high-risk mission-critical systems will likely need more stringent thresholds with differing weights for components, or a much more extended set of artifacts. Selecting, tuning, reading, and justifying those profiles requires domain knowledge and calibration of risk appetite. The scoring criteria in this governance framework are developed with a contextually-appropriate risk lens. In certain domains, a system-wide audit across all system levels will be necessary, which takes place outside the model boundary of practical data governance, runtime monitoring, incident response, and change control, to name a few. Critical safety-blockers such as fairness gaps, PII leakage, and residual risk capture familiar failure modes, but not all possible failure modes. They should be considered as an ever-changing set of restrictions and, when established, should continue to tighten or extend as new risks develop and jurisdictional expectations shift. Versioned policy enables the evolution of safety blockers but depends on timely development and curation with some form of governance.

AI governance is progressing toward an operational assurance direction that is comparable across organizations and continuously revisitable after model release. One visible trend is the emergence of structured, standardized reporting expectations for organizations developing advanced AI systems, such as shown in the G7 Hiroshima AI Process reporting framework (OECD, 2024) and the OECD's (OECD, 2025) subsequent monitoring-oriented initiative to support consistent disclosures. The UK AI Safety Institute (UK AI Safety Institute, 2024) has taken initiatives to formalize the evaluations focused on safety-related capabilities and limitations of AI. Also, the increasing use of capability and risk thresholds linked to evaluation requirements and mitigation actions prior to deployment is another approach that is proposed by Frontier AI Safety Commitments (UK Government, 2024), METR Common Elements (Model Evaluation and Threat Research (METR), 2024), and FMF Components (Frontier Model Forum, 2024). In parallel, the governments are also focusing on operationalising AI governance in the public sector through continous oversight and risk management directives (Office of Management and Budget, 2024), for safe development and deployment of AI systems (Department of Industry, Science and Resources (Australia), 2024). Audit-as-Code is designed to align with these trends by translating governance expectations into repeatable, machine-checkable evidence and deterministic gate decisions integrated into engineering workflows.

## 6 Conclusion

This study introduces the Audit-as-Code framework that maps and transforms AI governance from a descriptive set of guidelines into an executable, quantifiable, and verifiable checklist with deterministic outcomes and practical fixes. This framework caters to traceability and explainability indices that deal with a governance risk view to derive a quantifiable assured readiness score (ARS). Unlike the traditional documentation-driven approaches, Audit-as-Code technically maps verifiable artifacts like versioning, logging, decision trails, replication, explanation quality, and safety probes to explicit governance tiers and thresholds. And provides decisions with minimal and actionable Fix-It remedies. When evaluated, the Audit-as-Code framework demonstrated three core capabilities. Firstly, it integrates the deterministic eligibility criteria defined by TI/XI thresholds and enforces strict safety blockers. Secondly, it produces a defined, concrete, actionable set of fixes and remedies explicitly mapped to missing required documentation and record-keeping requirements. It also provides explanation coverage, post-processing ethical fairness checks, PII leakage detection, and other guardrails that help engineering and auditing teams to deploy and continuously monitor AI systems in a reliable and responsible manner. Lastly, it is designed to be portable and applicable across jurisdictional requirements. The changes in regulatory overlays may shift risk posture and readiness bands, but in no case does it reduce the minimum level of assurance that is needed for responsible deployment of an AI model.

## Data Availability

The original contributions presented in the study are included in the article/supplementary material, further inquiries can be directed to the corresponding author.
